# Ethephon Activates the Transcription of Senescence-Associated Genes and Nitrogen Mobilization in Grapevine Leaves (*Vitis vinifera* cv. Riesling)

**DOI:** 10.3390/plants10020333

**Published:** 2021-02-09

**Authors:** Maximilian Hendgen, Stefan Günther, Sven Schubert, Otmar Löhnertz

**Affiliations:** 1 Department of Soil Science and Plant Nutrition, Hochschule Geisenheim University, Von-Lade-Str. 1, D-65366 Geisenheim, Germany; Otmar.Loehnertz@hs-gm.de; 2 Bioinformatics and Deep Sequencing Platform, Max Planck Institute for Heart and Lung Research, Ludwigstr. 43, D-61231 Bad Nauheim, Germany; stefan.guenther@mpi-bn.mpg.de; 3 Institute of Plant Nutrition (iFZ), Justus Liebig University, Heinrich-Buff-Ring 26-32, D-35392 Giessen, Germany; sven.schubert@ernaehrung.uni-giessen.de

**Keywords:** ethylene, transcriptome, leaf senescence, stress response

## Abstract

Nitrogen (N) remobilization in the context of leaf senescence is of considerable importance for the viability of perennial plants. In late-ripening crops, such as *Vitis vinifera*, it may also affect berry ripening and fruit quality. Numerous studies on the model plant *Arabidopsis thaliana* have confirmed an involvement of the plant hormone ethylene in the regulation of senescence. However, ethylene research on grapevine was mostly focused on its involvement in berry ripening and stress tolerance until now. To investigate the effect of ethylene on the initiation, regulation, and progress of senescence-dependent N mobilization in grapevine leaves, we treated field-grown *Vitis vinifera* cv. Riesling vines with 25 mM ethephon at the end of berry ripening. Ethephon induced premature chlorophyll degradation and caused a shift of the leaf transcriptome equivalent to developmental leaf senescence. The upregulated metabolic processes covered the entire N remobilization process chain, altered the amino acid composition in the leaves, and resulted in an average 60% decrease in leaf N. Our findings increase the fundamental knowledge about the initiation and manipulation of leaf N remobilization in perennial woody plants by ethephon. This offers a methodological approach to the targeted induction of senescence and thus to an improvement in the N supply of grapes.

## 1. Introduction

With more than 7.5 million hectares of cultivated cropland worldwide and an annual production of approximately 75 million tons of grapes (status 2017), *Vitis vinifera* is a significant permanent crop and therefore of interest for applied research [1]. Numerous studies dealing with the hormonal implications on grapevine physiology have been conducted, especially focusing on flowering, stress response, veraison, berry ripening, and the synthesis of secondary metabolites in berries [2,3,4,5]. However, not much effort has been undertaken in investigating the hormonal regulation of grapevine leaf senescence, although transcriptome studies have revealed a profound change in gene expression due to the initiation of this process [6].

After growth and maturity, senescence denotes the final developmental stage of a leaf’s lifecycle [7]. It is a complex, genetically controlled aging process in plants that is triggered either developmentally by leaf age, or prematurely by external environmental factors such as, for example, drought or nutrient stress [8,9]. The aim is a recycling of nutrients—mainly nitrogen (N)—from the leaf into storage organs, as nutrient acquisition is an energy-intensive process and nutrient recycling, therefore, represents an evolutionary advantage for plants [10,11]. Both the rapidity of leaf aging and the integration of environmental factors are controlled by phytohormones [8]. The initiation of the senescence process leads to major alterations in gene transcription, in particular to the upregulation of senescence-associated genes (SAGs), and in consequence to the degradation of chlorophyll, proteolysis, N remobilization and finally to the decay of the leaf cells [7,10,12].

Nitrogen remobilization during leaf senescence requires both the degradation of N-containing macromolecules (e.g., via proteases, nucleases) and the transformation of the degradation products to phloem-mobile transportation forms (mainly glutamine and asparagine) [13,14]. As the majority of remobilized N origins from chloroplast proteins (namely, *Rubisco* and *LHC* proteins), proteolytic pathways such as autophagy and the ubiquitin 26S proteasome complex are of paramount importance during senescence [13,15,16]. Due to the related loss of photosynthetic activity, free amino acids are consumed as an alternative respiratory substrate while recycling the amino group [13]. For this purpose, glutamate and aspartate are deaminated to be fed to the citric acid cycle, and the released ammonium is re-assimilated to glutamine and asparagine to be translocated via the phloem [13]. An increased transcription of the N metabolism enzymes glutamate dehydrogenase (GDH) and cytosolic glutamine synthetase (GS1), as well as an increasing ratio of glutamine to glutamate, is characteristic for this transformation [17]; the same applies for the asparagine: aspartate ratio [18].

In perennial woody plants such as grapevine, N mobilization during leaf senescence is on the one hand of crucial importance for the nutrient refilling of storage organs at the end of the growth season [11]. On the other hand, the chloroplast destructive nature of leaf senescence can be deteriorating for the ripening process of high sugar accumulating fruits, such as grapes, when initiated too early [19]. Nevertheless, fruit ripening and leaf senescence often partly overlap in late-ripening varieties such as *Vitis vinifera* cv. Riesling when grown in a cool climate. Under such conditions, remobilized leaf N may have a positive effect on the storage of nitrogenous compounds such as amino acids and aroma precursors in the grape and thus contribute to an improvement in must fermentability and wine aroma [20]. Substantial amino acid depositions into the berry at the end of the ripening phase have already been documented for various grape varieties [21], but scientific research of a possible connection with N remobilization from the leaf is hampered by the large vintage-dependent divergence in the timing of ripening and senescence. Therefore, knowledge about the hormonal regulation of senescence in grapevine would open up new possibilities for a targeted induction of N-translocation, as well as its investigation with regard to a viticultural utilization to increase grape quality.

From other studies, mainly on the model plant *Arabidopsis thaliana*, the phytohormone ethylene is well known to have a pivotal role in the hormonal control of senescence initiation [22,23,24,25]. To investigate the relation between ethylene and plant physiology, expression analysis of the ethylene biosynthetic related transcripts and enzymes, ethylene level monitoring during plant development, mutant or transgenic plant studies, as well as exogenous ethylene application, are widely used techniques [26]. For the latter, the chemical substance (2-chloroethyl) phosphonic acid (ethephon) is an appropriate substitute for direct ethylene fumigation in field experiments, as it can be applied liquidly and releases ethylene at a defined dose after absorption by the plant cell [27].

Regarding grapevine, such surveys have already revealed an involvement of ethylene signaling in shoot growth [28], flowering [29], pathogen defense [30], the timing of veraison [4], and berry ripening [31]. Compared with this, there is only sparse knowledge about the role of ethylene in the activation of leaf senescence and N remobilization in grapevine. Even though some publications already reported about premature leaf yellowing as well as fruit and leaf abscission due to an ethylene treatment (as reviewed by Szyjewicz et al. [32]), none of them addressed the effects at a molecular level.

Hence, we hypothesized that an increase in ethylene triggers senescence and thereby N mobilization in leaves of *Vitis vinifera* cv. Riesling. To test this hypothesis, we treated *Vitis vinifera* cv. Riesling vines with ethephon in a field experiment and investigated its impact on senescence symptoms, such as chlorophyll degradation and SAG transcription, with a focus on the genetic and metabolic key points of N remobilization. The aim of our work was not only to provide fundamental insights into the initiation of leaf senescence in vines, but also to open the way to further research into the viticultural benefits of a targeted senescence induction. This could possibly optimize the ripening process of grapes and increase the number of nitrogenous compounds contained in the grapes.

## 2. Results

### 2.1. Chlorophyll Degradation

**Figure 1:**
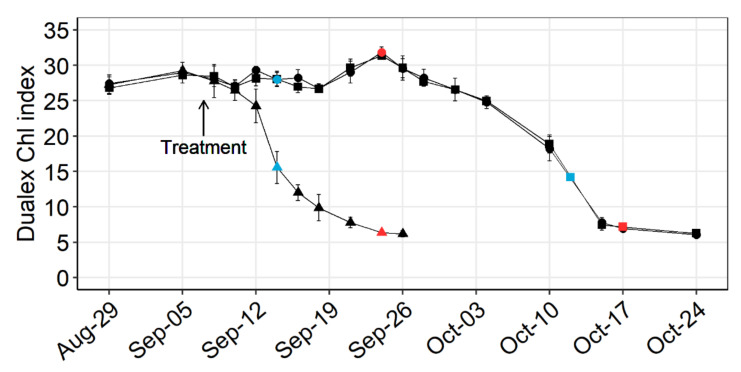
Time course of the Dualex Chl index of *Vitis vinifera* cv. Riesling leaves: The experimental treatments are control (●), ethephon treatment (▲) and natural senescence (■). The mean values and standard deviations of the three treatments are shown (*n* = 4). Sampling events for RNA sequencing and amino acid analysis are marked in blue, those for leaf N analysis in red.

The ethephon treatment caused premature chlorophyll degradation in the leaves (Figure 1). From the fifth day (Sep-12) after the treatment onwards, the Chl index of the ethephon-treated vine leaves was significantly lower than the readings of the control leaves (*p* < 0.05). The Chl index of the ethephon-treated leaves steadily dropped over 14 days until premature leaf fall on Sep-26. The Chl index of the control leaves started to decline due to developmental leaf senescence about three weeks later than the ethephon-treated leaves. Degradation rate and duration of the discoloration process were comparable between ethephon-treated and naturally senescing leaves.

### 2.2. Gene Transcription Analysis

**Figure 2:**
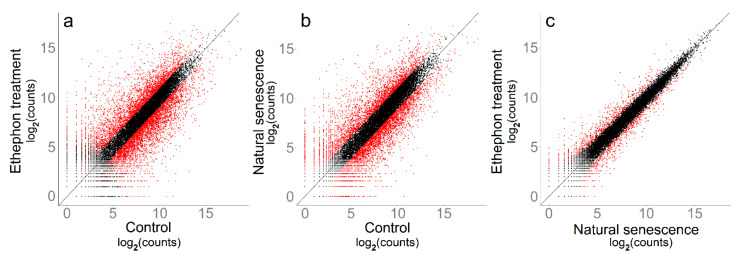
Scatter plots of log_2_-normalized mean gene counts of control vs. ethephon treatment (**a**), control vs. natural senescence (**b**) and natural senescence vs. ethephon treatment (**c**). Differentially transcribed genes (DTGs) of each pairwise comparison are marked red.

The RNA-Seq reads were assigned to 17,980 of a total of 30,434 *Vitis vinifera* genes. Pairwise treatment comparisons revealed a multitude of significantly up- and downregulated genes for ethephon treatment vs. control, as well as for natural senescence vs. control (Figure 2). Contrasting ethephon treatment vs. control, 7821 genes (43.5% of all assigned genes) were significantly differentially transcribed, the differentially transcribed genes (DTGs) consisted equally of up- and downregulated genes over the whole range of transcription rates (Figure 2a). Natural senescence vs. control appeared highly similar (Figure 2b), with a total of 7394 DTGs (41.2% of all genes). In contrast, the pairwise comparison ethephon treatment vs. natural senescence showed only 1333 DTGs (7.4% of all genes), and the majority of DTGs had a rather low transcription rate (Figure 2c).

**Figure 3:**
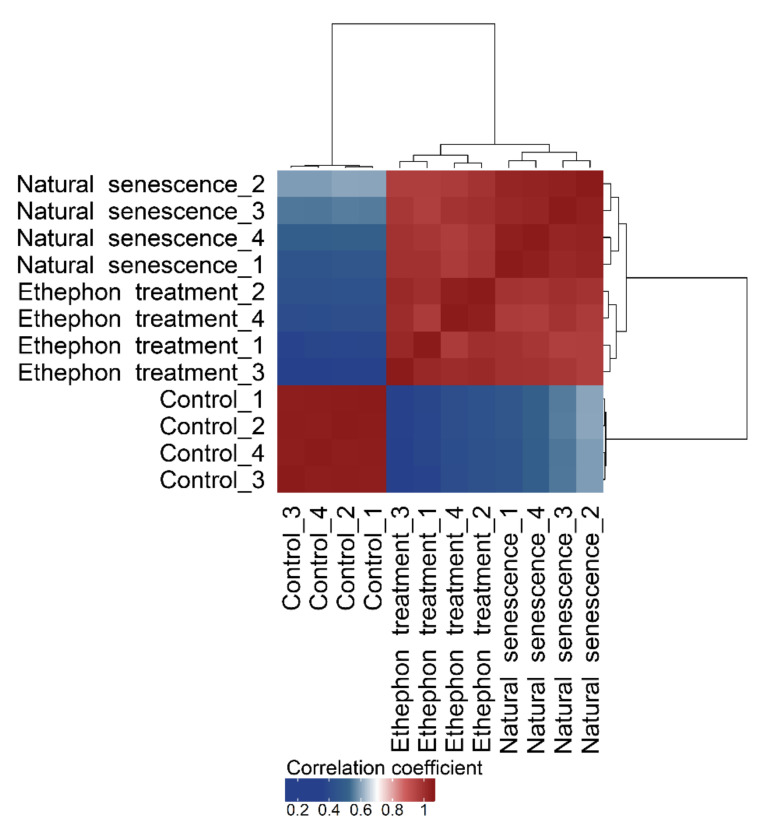
Hierarchically clustered correlation heatmap of control, ethephon treatment, and natural senescence sample transcriptomes. The color code shows Pearson’s correlation coefficient between the gene transcription profiles (normalized counts of 17,980 genes per sample) of all samples. The samples were clustered according to their Euclidean distances given by the dendrogram.

To check the sample transcriptomes for within- and inter-treatment similarity, Pearson’s correlation coefficients were calculated, and samples clustered according to their Euclidean distances (Figure 3). The clustering grouped the samples into two main clusters, with one cluster consisting of the ethephon treatment and natural senescence and the other cluster consisting the control (mature green leaf) samples. Within the ethephon treatment/natural senescence cluster, samples were grouped together by treatment. Pearson’s correlation coefficients between sample transcriptomes were high (0.917–0.997) within both main clusters, whereas the correlation between samples belonging to different clusters was uniformly low (0.391–0.608). The dendrogram also reflected this, showing a high Euclidean distance between the two main clusters compared to rather low distances among samples within the two clusters. Within the three experimental treatments, the ethephon treatment and the natural senescence showed greater Euclidean distances (thus variability) among their replicates than the control samples.

As the upregulation of senescence-associated genes (SAGs) is an essential characteristic for the activation of leaf senescence, the samples were checked for the transcription of a set of common molecular markers (Table 1). Of the selected SAGs, four were associated with transcriptional regulation (*NAC029*, *WRKY53* and *NAM*, *EIN3*), two with cell protection (*SAG13*, *MT*), two with proteolysis (*SAG12*, *Ubiquitin transferase*) and two with N mobilization (*GDH*, *GS1*).

The transcription of all ten SAGs was significantly increased in the ethephon-treated and the naturally senescing leaves compared to the control leaves (Table 2). No statistical difference was observed between the ethephon treatment and natural senescence in the transcription of *NAC029*, *EIN3*, *SAG12*, *Ubiquitin transferase*, and *GS1*. By contrast, the transcription level of *WRKY53*, *SAG13*, *MT*, and *GDH* was more elevated in the ethephon-treated leaves than in the naturally senescing ones. The only SAG with a significantly higher transcription rate in the naturally senescing leaves than in the ethephon-treated ones was *NAM*.

### 2.3. Pathway Analysis

**Figure 4:**
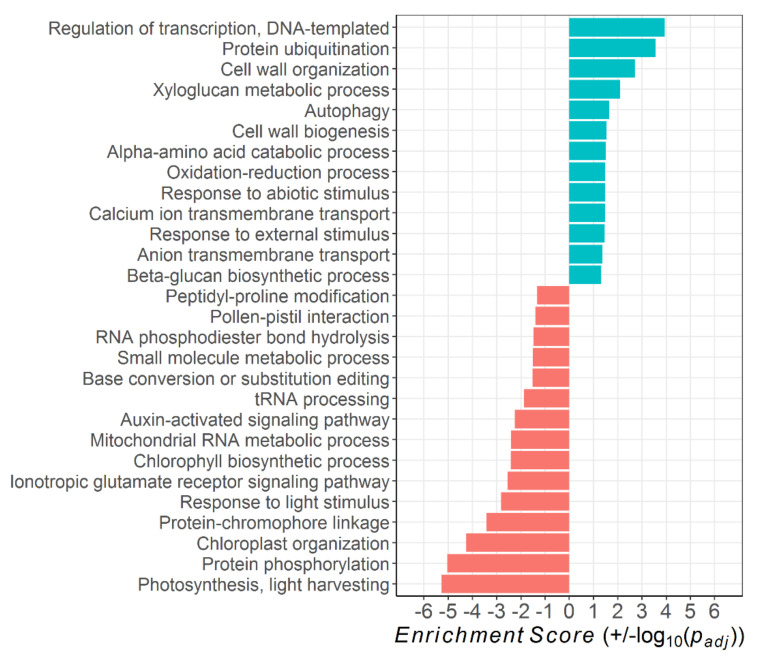
Significantly up- and downregulated (*p_adj_* < 0.05) *Gene Ontology* biological processes in leaves of ethephon-treated vs. control *Vitis vinifera* cv. Riesling vines. The *Enrichment Score* gives the log_10_ of the *p_adj_*-value according to *Fisher’s Exact Test*.

The DTGs of ethephon treatment vs. control belonged to 28 significantly altered biological processes, of which 13 were up- and 15 downregulated in the ethephon-treated leaves (Figure 4). While transcriptional regulation, protein ubiquitination, autophagy, amino acid catabolism, and response to external/abiotic stimulus were among the upregulated processes in the ethephon-treated leaves, a large proportion of their downregulated processes related to photosynthetic activity (namely, photosynthesis, chloroplast organization, protein–chromophore linkage, response to light stimulus, and chlorophyll biosynthetic process). In addition, protein phosphorylation and the ionotropic glutamate receptor signaling pathway were downregulated with high significance.

Color code: blue = upregulated in ethephon-treated leaves (+)

red = downregulated in ethephon-treated leaves (−)

**Figure 5:**
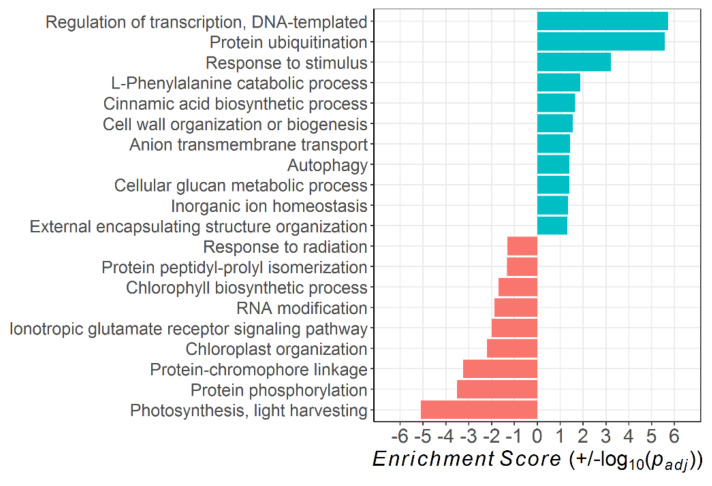
Significantly up- and downregulated (*p_adj_* < 0.05) *Gene Ontology* biological processes in leaves of naturally senescing vs. control *Vitis vinifera* cv. Riesling vines. The *Enrichment Score* gives the log_10_ of the *p_adj_*-value according to *Fisher’s Exact Test*.

Annotating the DTGs of natural senescence vs. control leaves resulted in 20 significantly affected biological processes, of which eleven showed an increase and nine a decrease during senescence (Figure 5). In accordance to the ethephon treatment, natural senescence led to increased transcriptional regulation, protein ubiquitination, autophagy, and response to stimulus, while photosynthesis, chloroplast organization, protein-chromophore linkage, response to radiation, chlorophyll biosynthetic process, protein phosphorylation, and the ionotropic glutamate receptor signaling pathway were downregulated. All in all, the effect of natural leaf senescence on pathway regulations highly resembles the one of the ethephon treatment.

Color code: blue = upregulated in naturally senescing leaves (+)

red = downregulated in naturally senescing leaves (−)

### 2.4. Nitrogen Remobilization

The concentrations of 16 of the 25 analyzed amino acids in total varied significantly between the treatments, most of them showing increased concentrations compared to the control leaves (Table 3). The concentrations of glutamine, histidine, isoleucine, leucine, methionine, phenylalanine, threonine, tryptophan, and valine were highest in the ethephon-treated leaves, followed by the naturally senescing leaves and lowest in the control leaves. The asparagine concentrations were increased in the leaves of the ethephon treatment, as well as during natural senescence, without a statistical difference between them. Arginine showed increased concentrations solely in the naturally senescing leaves, while the tyrosine and proline concentrations were solely increased in the ethephon-treated leaves. In contrast, the concentration of glutamate and of lysine was significantly lower in the ethephon-treated and naturally senescing leaves, compared to the control. Aspartate was reduced solely in the naturally senescing leaves. No treatment effect was detectable for alanine, citrulline, cystine, glycine, ornithine, serine, β-alanine, β-aminoisobutyric acid, and γ-aminobutyrate. The ammonium concentration in the leaves did not vary as well. In total, the ethephon-treated leaves showed an increased free amino acid concentration, whereas the control and the natural senescence were not distinguishable in the sum of their amino acids.

The ratios of glutamine:glutamate and of asparagine:aspartate were determined, as an increase in these ratios is indicative of amino acid respiration with simultaneous N remobilization during leaf senescence [13,15]. Both amino acid ratios increased significantly in ethephon-treated and naturally senescing leaves (*p* = 9.7e^−3^|*p* = 1.7e^−4^). In the ethephon-treated leaves, the mean glutamine:glutamate ratio had increased about 9-fold compared to the control leaves. In the naturally senescing leaves, the value increased 6-fold. The asparagine:aspartate ratio increased even stronger, the mean values of ethephon-treated and naturally senescing leaves exceeded the control by a factor of 25 and 32, respectively. No statistical difference was detectable between ethephon treatment and natural senescence.

**Figure 6:**
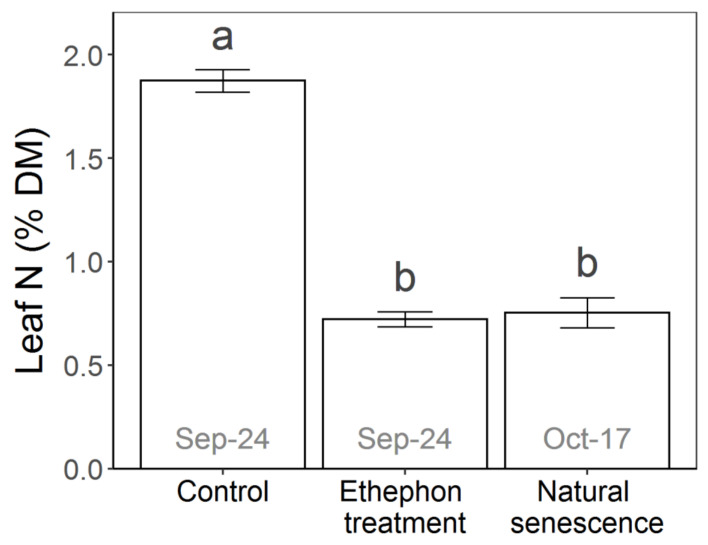
Mean total N concentration ± standard deviation in leaves of control, ethephon-treated and naturally senescing *Vitis vinifera* cv. Riesling vines. Leaves were sampled on two different dates as stated at the bars, at complete yellowing of the ethephon-treated (control and ethephon treatment) and at complete yellowing of the naturally senescing leaves. Experimental treatments with different letters on top differ significantly with *p* < 0.05.

The ethephon treatment, as well as natural senescence, caused a significant reduction in total leaf N concentration (*p* = 0.023) (Figure 6). No difference was detectable between the ethephon-treated (mean 0.75% of DM) and the naturally senescing leaves (mean 0.72% of DM) (*p* = 0.882), when sampled after completion of their chlorophyll degradation (Figure 1). The average N decline amounted to −59.8% in the ethephon-treated leaves and −61.4% in naturally senescing leaves, compared to the green control leaves (mean 1.87% of DM).

## 3. Discussion

Treating *Vitis vinifera* cv. Riesling vines with 25 mM ethephon at the end of the berry ripening phase caused premature leaf yellowing and early leaf fall. The visible symptoms were accompanied by an extensive remobilization of leaf N (Figure 6). With regard to the RNA sequencing, the ethephon-treated leaves not only showed a similar quantity of transcriptionally regulated genes compared to the control, their expression profile also highly correlated with that of naturally senescing leaves three weeks later (Figure 3). As the transcriptome of senescing grapevine leaves proved to be highly distinctive from other phenological states [6], our findings confirm a senescence-activating effect of ethylene in *Vitis vinifera* cv. Riesling.

Major shifts in gene expression during senescence have been reported by other researchers [6,41], the massive transcriptome alterations of the ethephon-treated and the naturally senescing grape leaves are therefore in accordance with these reports. Both treatments had a higher transcriptome variability between replicates compared to the control (Figure 3); the cause of this was presumably an increase in inhomogeneity within and between sampled leaves. A lack of consistency in the rates of senescence within the leaf—and even more between leaves—is a common problem in molecular senescence studies [14]. In addition, induction-specific alterations in the gene expression patterns of senescence have repeatedly been reported [42,43,44]. Such singularities in gene transcription are conceivable for ethylene-induced compared with developmental grapevine leaf senescence as well. As the vast majority of their DTGs was, however, only at a low transcription level and with minor count differences (Figure 2c), a high degree of analogy between these two types of leaf senescence in grapevine can nevertheless be confirmed.

All of the investigated SAGs were significantly higher transcribed in both the ethephon-treated and naturally senescing leaves compared to the control (Table 2). *Senescence-associated gene 12* (*SAG12*), which encodes a cysteine protease, is the most commonly used molecular marker for leaf senescence, as its upregulation is a specific response to natural leaf senescence [13]. In *Arabidopsis*, *SAG12* was only upregulated during age-dependent but not during ethylene-induced senescence [37]. Our findings with grapevine are contrasting this, as both ethephon-treated and naturally senescing leaves showed a substantial increase in *SAG12* transcription rate (Table 2).

*No apical meristem* (*NAM*) proteins belong to the *NAC*-type transcription factors and are involved in shoot apical meristem development, stress tolerance, senescence initiation and nutrient translocation [45,46]. Comparing to *SAG12*, an upregulation of *NAM* was observed in *Arabidopsis* solely during natural but not during dark-induced senescence [14]. In addition, its increase in transcription turned out to be dependent on functioning ethylene and jasmonate signaling [14]. However, in our study, the increase in *NAM* transcription of the naturally senescing leaves exceeded the ethephon treatment, despite the activation of the ethylene signaling pathway via ethephon.

By contrast, the transcription of *WRKY53*, *senescence-associated gene 13* (*SAG13*), *MT* (encoding a metallothionein protein), and *glutamate dehydrogenase* (*GDH*) was significantly higher in the ethephon-treated than in the naturally senescing leaves. The transcription factor *WRKY53* is assigned to be an early molecular marker for senescence, and its upregulation was shown to be age-dependent at whole-plant level in *Arabidopsis* [34,47]. While the developmentally senescing leaves confirmed this age-dependent response, its even higher upregulation in the ethephon-treated leaves compared to the control suggests also an age-independent, but ethylene-dependent, mode of action. *SAG13* (encoding an unspecific reductase) and *MT* are both involved in stress response, detoxification and cell protection during senescence [7,36]. As ethylene signaling interacts with many other plant hormones and therefore also plays a role in the control of stress response and environmental adaptation of plants [48], its consistently high expression in the ethephon-treated leaves might be a side-effect of the artificially enhanced ethylene signaling.

The transcription factor EIN3 (ETHYLENE-INSENSITIVE3) is a key player in the ethylene signaling pathway, and its transcription rate was found to increase with ageing in older leaves, increasing their sensitivity to ethylene and therefore promoting senescence [35]. *EIN3* was further assigned an important convergence point between age-dependent and environmentally induced senescence [35]. Our results are in accordance with these findings, as *EIN3* levels were increased in both ethephon-treated and naturally senescing leaves, thereby underlining the importance of ethylene signaling for the progression of natural senescence in grapevine leaves.

The results of the *pathway* analysis underline the high degree of similarity between ethylene-induced and developmental leaf senescence in *Vitis vinifera* cv. Riesling (Figure 4 and Figure 5). In both the ethephon-treated and the naturally senescing leaves, various photosynthesis-related processes were downregulated, while transcriptional regulation, autophagy, ubiquitination, responses to stimuli, and transmembrane transport processes were significantly upregulated. All these processes are congruent to findings on the annual plant *Arabidopsis thaliana* [41].

The upregulation of autophagy and protein ubiquitination is essential for proteolysis-based N mobilization during leaf senescence. An upregulation of several genes of the ubiquitin 26S proteasome proteolytic pathway has been proved for *Arabidopsis thaliana* and *Nicotina sylvestris* [38,49,50]. Autophagy is involved in the breakdown of *Rubisco* and other macromolecules, as well as in maintaining cell functioning during senescence [13,51]. For the subsequent translocation of proteolytically mobilized N, GDH and the cytosolic GS1 are key enzymes. GDH deaminates glutamate, the resulting 2-oxoglutarate is channeled to the citrate cycle for respiratory use. The released ammonium is re-assimilated into glutamine by GS1 and subsequently outsourced via the phloem [39,40]. An upregulation of *GDH* and *GS1* during leaf senescence has been proved for *Arabidopsis thaliana*, *Oryza sativa*, and *Nicotina tabacum* [14,17,40]. In this study, the leaves of the ethephon-treated and the naturally senescing vines showed an increased transcription of both genes, too.

In accordance with the upregulated transcription of *GDH* and *GS1*, both the ethephon-treated and the naturally senescing leaves showed an elevated glutamine and a reduced glutamate concentration. In combination, this resulted in the increased glutamine:glutamate ratio. The same applies for the likewise increased asparagine:aspartate ratio, which follows an equivalent amino acid conversion process during leaf senescence in *Arabidopsis* [18]. In terms of other amino acid changes, both experimental treatments consistently showed an increase in branched chain (leucine, isoleucine, valine) and aromatic amino acids (phenylalanine, tryptophan, tyrosine). The same result was found in a spatiotemporal metabolism study on *Arabidopsis* during developmental leaf senescence [18]. Additionally, the senescing Arabidopsis leaves also had higher levels of the stress response amino acids, such as proline, β-alanine, and γ-aminobutyric acid [18]. By contrast, no increase in stress response of amino acids was detectable in the control-late leaves, and the ethephon-treated leaves solely exhibited a higher proline level. As proline metabolism is expected to be affected by ethylene [52], the production of proline may have been stimulated by the ethephon treatment. However, as no accumulation of stress response amino acids in the developmentally senescing grape leaves occurred, *Vitis vinifera* cv. Riesling seems to deviate from *Arabidopsis thaliana* in this regard.

The last step of the N remobilization process during leaf senescence is the translocation of the released N out of senescing leaves via the phloem in the form of amino acids. Both the ethephon-treated and the naturally senescing grape leaves did not only accumulate glutamine and asparagine, which are the main amino acids in phloem sap during senescence [53], they also showed a substantial decline in total leaf N compared to the mature control leaves (Figure 6), thereby proving the removal of mobilized N. Their reduction in leaf N of around −60% was in accordance with results obtained on *Vitis vinifera* cv. Pinot noir in Oregon, USA [54]. *Vitis vinifera* sp. thereby seems to be at the upper limit for N remobilization of perennial plants, compared to findings on other perennials whose proportions ranged between 40 and 55% [55].

Taken together, the ethylene-induced leaf senescence mirrored developmental senescence in terms of chlorophyll degradation, whole transcriptome profile, activation of SAGs, and regulated metabolic processes. The consequence was an equally comprehensive remobilization of leaf N, although some minor amino acid variations between ethylene-induced and developmental leaf senescence occurred. Hence, we can accept the hypothesis that ethylene can activate leaf senescence and N mobilization in *Vitis vinifera* cv. Riesling. This result opens up the possibility for future research work to assess the importance of N retranslocation from senescing leaves for N storage into the grape berry. In the long term, precise control of nutrient translocation processes in grapevine could optimize fruit quality, increase nutrient use efficiency, and save fertilizer.

## 4. Materials and Methods

### 4.1. Experimental Site

The field experiment was conducted at a vineyard in the Rheingau wine region in Germany (49°58′51.4″ N 7°57′02.7″ E). It had been planted in 1986 with *Vitis vinifera* cv. Riesling grafted on *Vitis berlandieri* × *Vitis riparia* cv. SO4 at a spacing of 2.6 m^2^ per vine. The trellis system was a vertical shoot positioning (VSP), orienting east to west. The vineyard management followed the code of good practice in terms of integrated pest management and under-vine herbicide use. The inter-rows were permanently greened, and no fertilization had been applied since planting.

In 2018, some of the plants (one third) were treated with ethephon (25 mM) together with 0.1% (*v*/*v*) wetting agent (Adhäsit, Spiess-Urania, Hamburg, Germany) to improve the dispersion on the leaf surface. The application was performed about two weeks before harvest at around 22° Brix of the berries, corresponding to the phenological stage E-L 38 [56]. The canopy was sprayed from both sides until runoff. Leaf samples were taken from ethephon-treated and control vines seven days after the treatment, and additionally from control vines undergoing natural senescence 35 days after the treatment. Thereby, the effects of the ethephon treatment on gene transcription and nitrogen mobilization within the leaf could be compared to mature green leaves (control), as well as to developmentally senescing vine leaves (natural senescence). The experiment was set up as a completely randomized block design in which each of the three experimental treatments (control, ethephon treatment, natural senescence) was replicated in four blocks. Each block consisted of two neighboring row segments, each 15 vines in row.

### 4.2. Chlorophyll Monitoring

Leaf chlorophyll *a* + *b* was assessed using the handheld optical leaf clip sensor Dualex (Force-A, Orsay, France). The measurement is based on the differential transmission of light at two different wavelengths (710 vs. 810 nm) through a leaf minus a blank value and results in a dimensionless index between 0 and 150 [57]. The Dualex Chl index highly correlates with the chlorophyll and leaf N concentration of grapevine leaves, especially during ripening and senescence [58]. Readings were performed on the terminal blade of primary leaves positioned between rank 5 and 7, and each leaf was measured from the adaxial and abaxial side. For each block, the measured data from 12 leaves equally distributed from both sides of the canopy were averaged.

### 4.3. Leaf Sampling and Assessment of Nitrogen Parameters

For amino acid analysis and RNA sequencing, four primary leaves (rank 5 to 7) were sampled without petiole, immediately frozen in liquid nitrogen and stored at −80 °C until further processing. The control and the ethephon treatment were sampled on September 14th and the natural senescence treatment on October 12th, with chlorophyll concentration comparable to that of the ethephon treatment at the time of sampling.

Amino acids were extracted from 200 mg of ground leaf tissue with 2 mL buffer solution (96% (*v*/*v*) lithium citrate (40 mM, pH 2.2, Sykam, Fürstenfeldbruck, Germany) and 4% (*v*/*v*) norleucine (2.5 mM) as internal standard) for 30 min in an ice-cooled ultrasonic bath. The extract was centrifuged (15 min, 3200× *g*) and the supernatant transferred through a syringe filter (0.45 µm) into a 2 mL HPLC vial and sealed. The measurement was performed photometrically at 570 and 440 nm after chromatographic separation and postcolumn derivatization with ninhydrin using an amino acid analyzer (Aracus, MembraPure GmbH, Hennigsdorf, Germany).

For total leaf N analysis, ten primary leaves (rank 5 to 7) per block were sampled without petiole, washed and dried at 65 °C until constant weight. The control and the ethephon treatment were sampled on 24 September and the natural senescence variant on 17 October, after completion of the respective chlorophyll removal in the yellowing leaves. Dry leaves were ground with a centrifugal mill (Cyclotec, Foss, Hillerød, Denmark). Mineral N extraction and analysis were performed according to Friedel et al. [58].

### 4.4. RNA Isolation, Library Preparation, and Sequencing

Leaf total RNA was extracted from 100 mg ground leaf tissue using the Spectrum Plant Total RNA Kit (Sigma-Aldrich, St.-Louis, MO, USA), following the manufacturer’s protocol inclusive an on-column DNase digestion (On-Column DNase Digestion Set, Sigma-Aldrich, St.-Louis, MO, USA). RNA integrity was checked with a Fragment Analyzer (Agilent, Santa Clara, CA, USA) and libraries were prepared using the VAHTS Stranded mRNA-seq Library Prep Kit (Vazyme Biotech, Nanjing, China) according to the manufacturer’s instructions. The final libraries were equimolarily pooled and used for 75 bp single end sequencing on an Illumina NextSeq 500 (Illumina, San Diego, CA, USA), yielding in approximately 25 million reads per sample.

### 4.5. Bioinformatic Processing and Statistical Data Analysis

After quality checking (*FastQC*, Illumina, San Diego, CA, USA), the reads adapter ends were removed using *Cutadapt* [59] and sequences were trimmed using *Trimmomatic* [60]. Edited sequences were mapped against the *Vitis vinifera* cv. Pinot Noir reference genome (NCBI, accession number PRJEA18785, cultivar PN40024) using *STAR* [61]. Counts per gene were computed using the *featureCounts* function [62] of the *Rsubread* package [63]. For each pairwise treatment comparison, counts were normalized and basemean, log_2_ fold change (log_2_FC), and *p*-value (including a false discovery rate (FDR) correction) were computed for each gene using the *R* package *DESeq2* [64]. Finally, genes with basemean ≥ 5, *log_2_ fc* ≥ 1/≤ −1 and *p*_adj_ ≤ 0.05 were defined as significantly differentially transcribed genes (DTGs).

Statistical analyses and plotting based on *R* [65] and its graphical user interface *RStudio* [66] complemented by the *R*-based WIlsON-app [67]. Results were checked for uniformity and normal distribution prior to statistical testing. Depending on the pre-test, analyses of variance were estimated using ANOVA or Kruskal–Wallis test, followed by Tukey’s HSD or pairwise Wilcoxon as post-hoc test.

### 4.6. Gene Ontology Enrichment Analysis

UniProt gene IDs of the DTGs were annotated against the GO Ontology database (release: 7 March 2019, Annotation data set: GO biological process complete), using the PANTHER overrepresentation test (release: 11 July 2019). The Vitis vinifera gene database of the PANTHER classification system served as reference [68] and the annotated biological processes were checked for significance using Fisher’s exact test including an FDR correction (*p* < 0.05). For visualization, the log_10_ of the adjusted p_adj_-values of the significantly regulated biological processes was computed, and up- and downregulated processes were distinguished by assigning them plus or minus.

## 5. Conclusions

An ethephon treatment of mature, field-grown *Vitis vinifera* cv. Riesling vines during the late ripening phase rapidly induced premature chlorophyll degradation, altered gene transcription, and resulted in N remobilization. The transcriptome data are in accordance with comparable studies on the model plant *Arabidopsis thaliana* in terms of SAG expression and the regulation of metabolic pathways during the progression of leaf senescence. Furthermore, the achieved effects highly resembled the ones observed during developmental senescence of untreated grape leaves three weeks later, thereby confirming a regulatory key role of ethylene in the timing of leaf senescence in grapevine. To what extent the senescence-inducing effect is time- and dosage-dependent is a question that needs further investigation. Our findings may not only lead to a better understanding of the regulation of leaf senescence in perennial woody plants, they may also enable the manipulation of leaf nutrient remobilization in order to improve grape nitrogen concentration and wine quality.

## Figures and Tables

**Table 1:** Overview of examined senescence-associated genes (SAGs) within the gene transcription analysis of *Vitis vinifera* cv. Riesling leaves.

SAG	Accession	Encoded Enzymes	Reference
*NAC029*	VIT_01s0026g02710	NAC-type transcription factor 29	[33]
*WRKY53*	VIT_17s0000g01280	WRKY-type transcription factor 53	[34]
*NAM*	VIT_19s0014g03300	*no apical meristem* protein	[14]
*EIN3*	VIT_13s0047g00250	ETHYLENE-INSENSITIVE3	[35]
*SAG13*	VIT_13s0019g02180	Troponine reductase	[36]
*MT*	VIT_08s0007g00330	Metallothionein protein	[7]
*SAG12*	VIT_18s0001g09990	Cysteine protease	[37]
*Ubiquitin transferase*	VIT_03s0063g02000	Ubiquitin transferase	[38]
*GDH*	VIT_16s0039g02720	Glutamate dehydrogenase	[39]
*GS1*	VIT_01s0011g02200	Glutamine synthetase-1	[40]

The accession number corresponds to the gene ID of the *Ensembl Plant Genome Annotation Project*^69.^

**Table 2:** Mean log_2_ gene counts ± standard deviation of the examined SAGs in leaves of control, ethephon-treated, and naturally senescing *Vitis vinifera* cv. Riesling vines (*n* = 4).

	Control (log_2_(Counts))			Ethephon Treatment (log_2_(Counts))			Natural Senescence (log_2_(Counts))		
*NAC029*	7.56	±	0.21 ^b^	12.37	±	0.25 ^a^	12.77	±	0.29 ^a^
*WRKY53*	3.84	±	0.42 ^c^	11.61	±	0.10 ^a^	10.79	±	0.28 ^b^
*NAM*	10.81	±	0.14 ^c^	11.50	±	0.07 ^b^	12.38	±	0.08 ^a^
*EIN3*	14.40	±	0.13 ^b^	15.25	±	0.28 ^a^	15.52	±	0.13 ^a^
*SAG13*	7.86	±	0.61 ^c^	13.84	±	0.17 ^a^	12.53	±	0.27 ^b^
*MT*	8.12	±	0.21 ^c^	11.73	±	0.58 ^a^	10.50	±	0.69 ^b^
*SAG12*	13.99	±	0.17 ^b^	16.89	±	0.12 ^a^	16.64	±	0.21 ^a^
*Ubiquitin transferase*	10.60	±	0.12 ^b^	11.50	±	0.12 ^a^	11.56	±	0.15 ^a^
*GDH*	4.17	±	0.25 ^c^	9.68	±	0.58 ^a^	8.36	±	0.31 ^b^
*GS1*	11.51	±	0.10 ^b^	12.85	±	0.25 ^a^	12.63	±	0.29 ^a^

Treatments with different superscript letters in the row are significantly different with *p* < 0.05.

**Table 3:** Mean amino acid and ammonium concentrations, as well as the glutamine:glutamate and asparagine:aspartate ratio ± standard deviations, in leaves of control, ethephon-treated and naturally senescing *Vitis vinifera* cv. Riesling vines (*n* = 4).

	Control (mg kg^−1^ DM^−1^)			Ethephon Treatment (mg kg^−1^ DM^−1^)			Natural Senescence (mg kg^−1^ DM^−1^)		
Alanine	50.02	±	8.36 ^a^	37.35	±	8.58 ^a^	33.24	±	10.63 ^a^
Arginine	1.39	±	1.11 ^b^	2.95	±	2.21 ^ab^	5.66	±	2.60 ^a^
Asparagine	0.74	±	0.91 ^b^	7.97	±	2.53 ^a^	8.83	±	1.51 ^a^
Aspartic acid	451.33	±	59.82 ^a^	330.91	±	74.54 ^ab^	283.44	±	28.31 ^b^
Citrulline	0.00	±	0.00 ^a^	0.63	±	1.27 ^a^	0.91	±	1.82 ^a^
Cystine	2.38	±	4.75 ^a^	11.52	±	5.11 ^a^	10.98	±	4.00 ^a^
Glutamic acid	765.06	±	89.73 ^a^	387.24	±	78.28 ^b^	394.34	±	28.42 ^b^
Glutamine	118.78	±	28.32 ^c^	531.32	±	26.44 ^a^	354.11	±	39.58 ^b^
Glycine	15.63	±	3.69 ^a^	17.57	±	2.10 ^a^	18.28	±	2.38 ^a^
Histidine	15.33	±	3.63 ^c^	57.07	±	3.08 ^a^	33.43	±	2.93 ^b^
Isoleucine	20.55	±	5.71 ^c^	74.14	±	3.61 ^a^	51.29	±	5.14 ^b^
Leucine	16.10	±	5.83 ^c^	83.22	±	7.41 ^a^	65.25	±	6.19 ^b^
Lysine	32.84	±	5.45 ^a^	15.83	±	6.57 ^b^	14.10	±	1.53 ^b^
Methionine	1.34	±	1.64 ^c^	23.37	±	2.81 ^a^	15.48	±	2.82 ^b^
Ornithine	0.00	±	0.00 ^a^	2.44	±	3.32 ^a^	3.08	±	2.22 ^a^
Phenylalanine	39.62	±	6.07 ^c^	76.19	±	8.44 ^a^	55.57	±	4.22 ^b^
Proline	7.93	±	1.47 ^b^	84.88	±	21.56 ^a^	35.84	±	15.75 ^b^
Serine	68.05	±	11.36 ^a^	57.79	±	6.92 ^a^	62.61	±	6.95 ^a^
Threonine	34.80	±	4.53 ^c^	80.65	±	7.06 ^a^	56.78	±	3.03 ^b^
Tryptophan	1.62	±	1.19 ^c^	95.37	±	19.08 ^a^	28.94	±	14.10 ^b^
Tyrosine	0.94	±	1.88 ^b^	11.93	±	7.91 ^a^	6.76	±	3.07 ^ab^
Valine	28.70	±	9.92 ^c^	99.65	±	9.96 ^a^	59.46	±	7.36 ^b^
β-Alanine	6.64	±	8.83 ^a^	9.63	±	3.24 ^a^	13.92	±	4.72 ^a^
β-Aminoisobutyric acid	1.48	±	2.96 ^a^	0.92	±	1.83 ^a^	0.00	±	0.00 ^a^
γ-Aminobutyric acid	24.54	±	3.74 ^a^	21.01	±	4.02 ^a^	20.69	±	2.48 ^a^
Total	1705.8	±	175.2 ^b^	2121.5	±	176.3 ^a^	1633.0	±	48.6 ^b^
Ammonium	17.89	±	0.89 ^a^	33.75	±	3.37 ^a^	14.76	±	17.89 ^a^
Glutamine:glutamate *	0.155	±	0.026 ^b^	1.421	±	0.336 ^a^	0.904	±	0.146 ^a^
Asparagine:aspartate *	0.001	±	0.002 ^b^	0.025	±	0.007 ^a^	0.032	±	0.007 ^a^

Treatments with different superscript letters differ significantly with *p* < 0.05. * The two amino acid ratios are dimensionless parameters.

## Data Availability

Sequence data are available in the short read archive (SRA) of the National Center for Biotechnology Information (NCBI), under accession numbers PRJNA643844.
